# Four New Cases of Hypomyelinating Leukodystrophy Associated with the *UFM1* c.-155_-153delTCA Founder Mutation in Pediatric Patients of Roma Descent in Hungary

**DOI:** 10.3390/genes12091331

**Published:** 2021-08-27

**Authors:** Zsuzsanna Szűcs, Réka Fitala, Ágnes Renáta Nyuzó, Krisztina Fodor, Éva Czemmel, Nóra Vrancsik, Mónika Bessenyei, Tamás Szabó, Katalin Szakszon, István Balogh

**Affiliations:** 1 Division of Clinical Genetics, Department of Laboratory Medicine, Faculty of Medicine, University of Debrecen, 4032 Debrecen, Hungary; szucs.zsuzsanna@med.unideb.hu; 2 Velkey László Child Health Center, Borsod-Abaúj-Zemplén County Central Regional Hospital and University Educational Center, 3526 Miskolc, Hungary; alatif.aker@gmail.com (R.F.); agnes.nyuzo@gmail.com (Á.R.N.); dr.fodor.krisztina.csilla@gmail.com (K.F.); 3 Neurodevelopmental Ward, St. Margaret Hospital, 1032 Budapest, Hungary; eva.czemmel@gmail.com; 4 Division of Radiology, Department of Medical Imaging, Faculty of Medicine, University of Debrecen, 4032 Debrecen, Hungary; vrancsiknora@med.unideb.hu; 5 Institute of Pediatrics, Faculty of Medicine, University of Debrecen, 4032 Debrecen, Hungary; besenyei.monika@med.unideb.hu (M.B.); szabotamas@med.unideb.hu (T.S.); szakszon.katalin@med.unideb.hu (K.S.); 6 Department of Human Genetics, Faculty of Medicine, University of Debrecen, 4032 Debrecen, Hungary

**Keywords:** *UFM1*, Roma founder mutation, ufmylation, Hypomyelinating leukodystrophy type 14, NGS

## Abstract

Ufmylation is a relatively newly discovered type of post-translational modification when the ubiquitin-fold modifier 1 (UFM1) protein is covalently attached to its target proteins in a three-step enzymatic reaction involving an E1 activating enzyme (UBA5), E2 conjugating enzyme (UFC1), and E3 ligase enzyme (UFL1). The process of ufmylation is essential for normal brain development and function in humans. Mutations in the *UFM1* gene are associated with Hypomyelinating leukodystrophy type 14, presenting with global developmental delay, failure to thrive, progressive microcephaly, refractive epilepsy, and hypomyelination, with atrophy of the basal ganglia and cerebellum phenotypes. The c.-155_-153delTCA deletion in the promoter region of *UFM1* is considered to be a founding mutation in the Roma population. Here we present four index patients with homozygous *UFM1*:c.-155_-153delTCA mutation detected by next-generation sequencing (whole genome/exome sequencing) or Sanger sequencing. This mutation may be more common in the Roma population than previously estimated, and the targeted testing of the *UFM1*:c.-155_-153delTCA mutation may have an indication in cases of hypomyelination and neurodegenerative clinical course in pediatric patients of Roma descent.

## 1. Introduction

Post-translational modification is a regulation process where enzymatic modification of proteins occurs during or after protein biosynthesis [1]. Ubiquitination is one type of more than 200 of these post-translational modifications, where ubiquitin molecules are attached to a protein as a result of a series of enzymatic reactions and serve as signal molecules. Therefore, modification by ubiquitin and ubiquitin-like proteins plays a significant role in several different cellular functions and signaling pathways [1,2]. One such ubiquitin-like protein is a 9.1-kDa protein with a tertiary structure similar to ubiquitin, the ubiquitin-fold modifier 1 (UFM1) protein encoded by the *UFM1* gene located on the forward strand of chromosome 13 [3,4]. The highly conserved process of covalently attaching UFM1 to its target proteins is called ufmylation, and just like the ubiquitination process, the ufmylation also consists of a three-step enzymatic reaction (E1-E2-E3). The UFM1 protein is activated by the E1 activating enzyme (UBA5) and forms a high-energy thioester bond, then the activated UFM1 is transferred to the E2 conjugating enzyme (UFC1), forming a similar thioester linkage. The last step of the ufmylation process is the transfer of UFM1 to its substrate protein, catalyzed by the E3 ligase enzyme (UFL1) [1,3,5].

Hypomyelinating leukodystrophy is a genetically heterogeneous disorder of the central nervous system in which myelin is not formed properly [6]. According to the OMIM (Online Mendelian Inheritance in Man) database, it has about 20 different types, of which Hypomyelinating leukodystrophy type 14 (MIM # 617899) is caused by mutations in the *UFM1* gene and is inherited in an autosomal recessive manner. It is characterized by hypotonia, an almost complete lack of motor or cognitive skills, absent language development, spasticity, and intractable seizures. Several patients also present with perceptive hearing loss and/or blindness, require tube feeding or ventilatory support, and most die in the first few years of life [7].

The process of ufmylation is essential for normal brain development and function in humans [5]. Mutations in the gene encoding proteins of the ufmylation cascade (*UBA5*, *UFC1*, *UFM1*) have been identified as causing severe early-onset encephalopathy with hypopmyelination [5]. Only two disease-causing mutations have been reported in the Human Gene Mutation Database (HGMD, Professional version 2021.2) in the *UFM1* gene so far, associated with global developmental delay, failure to thrive, progressive microcephaly, refractive epilepsy, and hypomyelination with atrophy of the basal ganglia and cerebellum phenotypes, and the same two mutations have been reported as pathogenic in the ClinVar database (Version accessed on 7 February 2021) associated with Hypomyelinating leukodystrophy type 14.

The *UFM1* (NM_016617.4, canonical transcript) c.-155_-153delTCA mutation, present in the promoter region of the gene, has the neural cell-specific effect of causing a significant reduction in transcription activity. 

In 2017, Hamilton et al. were the first to describe the homozygous *UFM1*:c.-155_-153delTCA founder mutation (reported as NM_001286704.1: c.-273_-271delTCA) in 16 patients with severe developmental delay, most of them having a Roma background with a high consanguinity rate. This deletion is reported to be a founder mutation in the Roma population, with a carrier frequency of 3–25%. Hamilton et al. reported an overall carrier rate of 4.5% when screening 670 Roma controls among different Roma communities across Europe, and a carrier rate of 25% in an endogamous community in Eastern Slovakia with a high rate of consanguinity [7]. The median age of the appearance of the first symptoms was 2 months. Every patient presented with seizures at 18 months at the latest, often with severe and drug-resistant epilepsy; all of them had microcephaly, absent speech, and their level of comprehension was limited to social awareness only. Ninety-four percent had no intentional movements and 81% presented with spasticity. MRI findings of these patients showed severe hypomyelination, putamen atrophy, and distinctive caudate nucleus abnormalities. All patients were at the most severe end of hypomyelination, with atrophy of the basal ganglia and cerebellum spectrum, and a median survival of 2 years, the most common cause of death being respiratory insufficiency. 

This homozygous 3-bp deletion in the promoter of the *UFM1* gene has further been described in other patients with AR Hypomyelinating leukodystrophy type 14, presenting with microcephaly, hearing impairment, seizures, and global developmental delay, co-segregating in affected sibling and detected by genome sequencing (reported as NM_001286704.1(*UFM1*):c.-273_-271del) [8] in Pakistani (reported as NC_000013.10 (NM_016617.2):c.-155_-153del) [9] and Slovenian patient(s) with progressive neurodegenerative disease (reported as NM_016617.2(*UFM1*):c.-155delTCA) [10]. The Slovenian patient had profound intellectual disability and epilepsy with a history of apnoeic episodes and feeding difficulties in infancy. Both parents had Roma ancestry, and several other members of the family had a similar clinical presentation.

This mutation is present in the HGMD Professional version 2021.2 (ID: CD1715768) and the ClinVar (Variation ID: 495149) mutation databases; it is absent from the gnomAD population database, and it is classified as a likely pathogenic based on ACMG guidelines [11].

## 2. Patients and Methods

### 2.1. Patients

Genetic testing of patients was performed solely for a diagnostic purpose, after obtaining written informed consent from the legal guardians, according to national regulations. Patient management was in accordance with the World Medical Association Declaration of Helsinki [12]. No patient selection based on ethnicity took place prior to testing; all patients seeking medical help with developmental delay/intellectual deficit with or without visible brain MRI anomaly were offered appropriate genetic tests.

### 2.2. Methods

Genomic DNA was isolated from peripheral blood leukocytes using the QIAamp Blood Mini kit (Qiagen GmbH, Hilden, Germany).

Targeted Sanger sequencing (patients 4, 7, 8, and 9) or Sanger confirmation of the NGS data (patients 1, 2, and 3) was performed in the case of every single patient. Sanger sequencing of the promoter region of the *UFM1* gene was performed using the BigDye Terminator v3.1 Cycle Sequencing kit (Applied Biosystems, Foster City, CA, USA) according to the manufacturer’s protocol using a 5′-ctagggtcctacgctctatgc-3′ forward primer and a 5′- ggggtagcacgacttcctct -3′ reverse primer.

Whole Exome Sequencing (WES) was performed on the Illumina NextSeq 500 sequencer system in 2 × 150 cycle paired-end mode. A Twist Human Core Exome kit (Twist Bioscience) (patient 3) or a Nextera DNA Exome kit (Illumina) (Patient 2) was used for library preparation.

Whole Genome Sequencing (WGS) was performed by Novogene (UK) Company Ltd., and raw data was sent back for analysis to our laboratory (Patient 1).

In the case of WES and WGS data, raw data were aligned to the hg19 reference genome using NextGene software (SoftGenetics, State College, PA, USA), the variant list was uploaded to the Franklin Analysis Platform (Genoox) [13] for variant classification, and HPO terms were used to help with variant prioritization.

In the case of every index patient, neuroimaging was performed at 4–8 months of age.

Clinical examination of Patients 1 and 2 was carried out on a 3T Achieva MR scanner (Philips Medical Systems, Koninklijke Philips N.V.). High-resolution 3D T1 weighted gradient echo (TR:8 ms; TE:3.8 ms; voxel size: 1 × 1 × 1 mm^3^) and T2 weighted turbo spin echo imaging (TR: 4000 ms; TE: 90 ms) were prepared for evaluation of anatomical structures and myelination. T1 fast field echo, FLAIR, SWI, and DWI measurements were also performed; these images are not presented in this study. 

Single voxel proton MR spectroscopy was made for Patient 2, with the following settings: TR: 2000 ms; TE: 144 ms; and voxel size: 1.5 × 1.5 × 1.5 cm^3^; the voxel was placed onto the white matter in the subinsular area based on the suggestions of Kugel et al., 1998, and Tahyyil et al., 2010 [14,15]. The other two babies were examined in another institute.

Patient 3 was examined on a Siemens Amira 1.5T unit (Siemens Healthcare GmbH, Germany). Without sedation, only DWI and T2 blade and T2 dark fluid measurements were successful.

An MRI scan of Patient 4 was made on Ingenia 3T equipment (Philips Medical Systems, Koninklijke Philips N.V.), 3D T1 weighted images (TR: 7.9 ms TE: 3.6 ms) and T2 weighted turbo spin echo imaging (TR: 5500 ms; TE: 100 ms), along with T1 fast field echo, SWI, and DWI measurements. A single voxel proton spectroscopy of the postcentral white matter sample area was made.

Anthropometric measurements were based on the child growth standards of Hungarian children, published by Joubert et al., 2006, Demographic Research Institute [16].

## 3. Results

### 3.1. Case Presentations

Here we present the clinical phenotypes of four index patients harboring the *UFM1*:c.-155_-153delTCA mutation in homozygous form (Table 1). All four patients were of Roma ethnicity. Table 2 describes the clinical features of the index patients.

#### 3.1.1. Patient 1

The patient was born as the first child of healthy parents in the 38th week of gestation with no knowledge of consanguinity. The mother had two healthy children from her previous marriage. The pregnancy and the perinatal period were uneventful. Birth weight was normal (25 pc); length and OFC were not recorded. Inspiratory stridor was recognized at two weeks of age, but it was not severe enough to prompt invasive investigations at that time.

At 5 months, frequent colic-like painful crying necessitated in-patient admission. Intestinal invagination was ruled out on ultrasonography. At this age, the motor performance, attention, and cognitive abilities of the patient showed remarkable delay—she did not try to roll over, did not pick up eye contact, her visual behavior suggested a problem in perception, and she had axial hypotonia and spasticity of the extremities. Routine laboratory tests, including serum ammonia and lactate, resulted negative. Repeated, intense crying fits, irritability upon physical stimuli turning promptly into stupor and lethargy remained unexplained. Tonic fits with the extension of the upper limbs raised the possibility of epilepsy, EEG showed a diffuse cortical functional abnormality, most detectable in the right fronto-centro-temporal areas. Brain MRI was performed and revealed brain atrophy. On follow-up examination, this was recognized as progressive global atrophy; delayed myelination also became obvious, along with the smaller caudate and lentiform nuclei. Lumbar puncture and CSF analysis resulted in normal cell count and glucose, but subnormal protein level (138 mg/L) (Ref: 200–400 mg/L). Biphasic, loud stridor was evaluated by laryngoscopy, which—other than a long epiglottis—revealed normal anatomical structures of the larynx and upper airways and suggested that an abnormal laryngeal tension due to impaired innervation was the likely cause of stridor. Bronchofiberoscopy detected normal lower airways. At 5 months, weight was 3 pc, length 3 pc, and OFC was 10–25 pc. At 1 year of age, weight was 1 kg < 3 pc, length 3 pc, and OFC was 10 pc. Unfortunately, there were no further precise measurements on the head circumference due to the patient’s condition requiring acute medical help upon admissions; only text-wise remarks on obvious microcephaly. At 6 months, deep apneas, bradycardia requiring intravenous atropine, bradypnea (8/min), and somnolence necessitated intensive care. The worsening of the respiratory failure required tracheostomy later on. Cultures taken from blood and CSF to identify an infectious origin of encephalopathy resulted negative. Dysphagia necessitated feeding via nasogastric tube and later via gastrotube. The patient was institutionalized and died at the age of 17 months. Before knowing the genetic diagnosis, the couple engaged in a further pregnancy, from which a healthy boy was born, who later was confirmed to be a healthy carrier of the *UFM1* variant.

#### 3.1.2. Patient 2

The patient was born in the 39th week of gestation with a weight at 25 pc and head circumference of 50–75 pc; perinatal adaptation was normal. The mother had three healthy children from her previous partner, one otherwise healthy boy with valvular pulmonary insufficiency and the affected proband from her second spouse. She had no knowledge of consanguinity. At one week of age, somnolence was noted by the general pediatrician and a clinical examination was requested. By 6 weeks of age, severe attention deficit and overall spasticity were obvious; the infant did not look at human faces and did not respond to visual stimuli, but he seemed to be able to track light. Ophthalmological examination described a pigment-ring around the papilla; flash-VEP detected no evoked potentials. At 3.5 months, microcephaly and overall but predominantly upper limb spasticity were noted. Dysphagia necessitated tube feeding. Bradypnea with a breathing rate of 8/min (at 13 months) was measured, and inspiratory stridor was noted. Tracheostomy and gastrotubes were inserted to ensure adequate calorie intake and to circumvene laryngeal dysfunction. There were descriptions of heart rate lability with a tendency to tachycardia, but no written numerical records were made. Tonic fits prompted EEG analysis, which revealed immature sleep patterns and diffuse cortical dysfunction dominated by theta waves while awake and delta waves while asleep. Baclofen was introduced. At 25 months of age, tonic-clonic fits necessitated repeated neurological examination and EEG, which revealed interictal epileptic spikes over the right hemisphere; therefore carbamazepine was introduced. At 4 months, weight was 1 kg < 3 pc, length 5 cm < 3 pc, and OFC 2 cm < 3 pc.

At 8 months, brain MRI showed decreased brain volume and severely delayed myelination (neither the semioval centrum nor the corona radiata showed myelination) with small, T2 hyperintense lacunas along the border of the grey and white matter, predominantly in the frontal brain and hypoplastic optic nerves. No visible cognitive or motor development was ever achieved. The patient died at 38 months from apneas, cardiopulmonary arrest, and fever without identifiable infection and normal inflammatory parameters.

#### 3.1.3. Patient 3

The patient was born as the sixth child of healthy parents; his two siblings had died of an unidentified condition, suspected to be a metabolic disease with a very similar clinical course. Parental consanguinity was never confirmed based on family reports. The patient was born in the 36th week of gestation with normal weight (90 pc); there were no records of the head circumference at birth. At 6 months, he was referred to a pediatric ward because of the positive family history and early signs of neurological disease, including poor visual attention and abnormal muscle tone. On physical examination, borderline microcephaly (3 pc), failure to thrive (weight at 0.8 kg < 3 pc, length 1 cm < 3 pc), spastic tetraparesis with upper limb predominance, clonisation of the lower limbs and exaggerated deep tendon reflexes, axial hypotonia, inspiratory stridor, and bradypnea were present. The latter had a peculiar pattern with “saccadic” inhalation (more than one shallow inhalation at a time and one exhalation). Swallowing and sucking inability necessitated nasogastric tube feeding. EEG revealed irregular, disorganized 100–150 µV 3–6 Hz delta-theta waves without spikes, suggesting encephalopathy. Ophthalmology revealed strabismus and minimal horizontal nystagmus during lateral gaze on both sides. Tandem mass spectrometry resulted negative (26 metabolites in frames of routine screening from dried blood spot); plasma ammonia was normal.

By 7 months of age, generalized hypotrophy and microcephaly (1 cm < 3 pc) were more remarkable; muscle spasms, excessive sweating, complete lack of attention to visual or auditory stimuli, bradypnea, and lack of basic motor skills necessitated long-term hospitalization. EEG showed severe cortical dysfunction with 90–100 µV 2–3 Hz delta waves with frontal theta waves. Brain MRI could only be performed without sedation, limited to T2 weighted, T2 dark-fluid, and diffusion-weighted axial sequences—these showed brain atrophy. Still, the smaller basal ganglia were recognizable.

One of the deceased siblings, a girl born to term with normal bodily parameters, showed severe psychomotor retardation, spastic tetraparesis, somnolence, congenital stridor, and dysphagia. Brain MRI showed a diffuse myelination defect. She lost contact with her surroundings at around 10 months of age and died of inspiratory failure at 2 years 6 months.

The other deceased sibling, also a female, was born to term with normal weight; microcephaly developed by 5 months of age. Spastic tetraparesis, loud inspiratory stridor with jugular retraction necessitated hospitalization. Visual attention was not provokable from this age. EEG showed subdelta-theta background activity of moderate amplitudes, mixed with temporo-parietal delta waves during apneas. Progressive neurological decline, tachycardia, and therapy-resistant profound apneas led to the patient’s death at 10.5 months of age.

#### 3.1.4. Patient 4

The patient was born as the second child of healthy parents who were second cousins. Birth parameters were normal (38th week of gestation, weight 3–10 pc, length 3 pc, OFC 50–75 pc, Apgar 9/10). The first child of the parents has situs inversus and normal intellect. Axial hypotonia and thermolability were noted at birth; one registered body temperature was 35.4 °C. Blood pressures were normal. Brain ultrasonography revealed triventricular dilatation and irregular gyrification. Brain MRI presented atrophy, but less severe callosal thinning. Delayed myelination and the characteristic appearance of basal ganglia was similar to the other above-described patients, but less advanced. At 3.5 months, a detailed neurological examination described axial hypotonia and spasticity of the limbs, especially the lower limbs, with positive myotatic reflexes, exaggerated deep tendon reflexes and clonisation on the lower limbs, and tremor on the upper limbs. Alertness and overall general activity were decreased, irritability and somnolence, as well as the circadian rhythm of sleep and wake periods, were diminished. Archaic reflexes were still provokable, and symmetric tonic neck reflex was exaggerated. Verticalization and locomotion were both severely impaired. Visual attention was hardly provokable, tracking human face or contrast table could be achieved for seconds only. Loud auditory stimuli resulted in a startle response; normal auditory stimuli could not provoke orientation or attention. The infant did not grab objects placed in her palm, nor did she reach for any. Psychomotor development was significantly delayed. At 4 months, Babinski and Rossolimo reflexes were still provokable; a social smile never developed. 

Visual evoked potential showed normal vision; Brain Evoked Response Audiometry could not detect hearing on either side. EEG showed hypersynchronous background activity. At 3.5 months, head circumference was 75 pc with bitemporal narrowing and a scaphocephalic shape of the skull.

### 3.2. MR Findings

**Figure 1:**
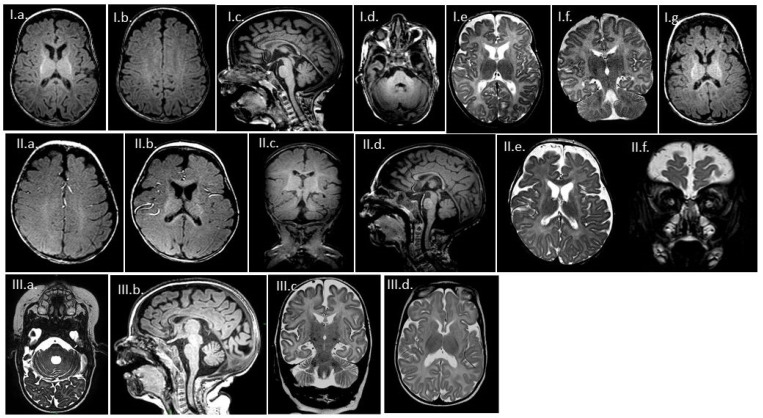
Brain MRI images of Patients 1, 2, and 4. Patient 1: T1 weighted (**I.a**–**d**) and T2 weighted (**I.e**,**f**) images at the age of 5 months and 8 months (**I.g**) on T1 sequence; Patient 2: T1 (**II.a**–**d**) and T2 (**II.e**,**f**) weighted images at 8 months of age; Patient 4: T2 weighted axial image at 4 months of age (**III.a**), T1 sagittal image (**III.b**), T2 coronal and axial planes (**III.c**,**d**).

MRI findings of index Patients 1, 2, and 4 are presented in Figure 1.

T1 weighted (Figure 1I.a–d) and T2 weighted (Figure 1I.e,f) images of Patient 1 at age 5 months shows normal gyrification but decreased brain volume. The caudate and especially the lentiform nuclei are small, suggesting atrophy of the basal ganglia (Figure 1I.a,e,f). The degree of myelination, based on the appearance of the basal ganglia and cerebellar peduncles, is similar to a newborn brain (Figure 1I.d). The upper zone of the corticospinal tracts shows absent myelination—at supraventricular level; the normally high T1 and low T2 signals, which should already be present at birth, are missing (Figure 1I.b,f). The corpus callosum is thin and does not show the bright signal of myelination (I.c). T2 weighted axial (Figure 1I.e), and coronal T2 fatsat (Figure 1I.f) show myelination only up to the level of the basal ganglia. At 8 months (Figure 1I.g), the atrophy showed progression, but the appearance of the white matter remained the same. 

T1 (Figure 1II.a–d) and T2 (Figure 1II.e,f) weighted images of Patient 2 at 8 months of age show a decreased volume of the brain, pronounced at the caudate and lentiform nuclei. White matter signals are most remarkably decreased in the perirolandic area (Figure 1II.a,c). The optic nerves are also thinner (Figure 1II.f). 

Thin medulla oblongata can be seen on the T2 weighted axial image of Patient 4 at 4 months of age (Figure 1III.a), along with a mildly thinner corpus callosum on a T1 sagittal image (Figure 1III.b) and general atrophy and small caudate nuclei on the T2 coronal and axial planes (Figure 1III.c,d).

All patients show secondary microcephaly and a scaphocephalic head shape.

**Figure 2:**
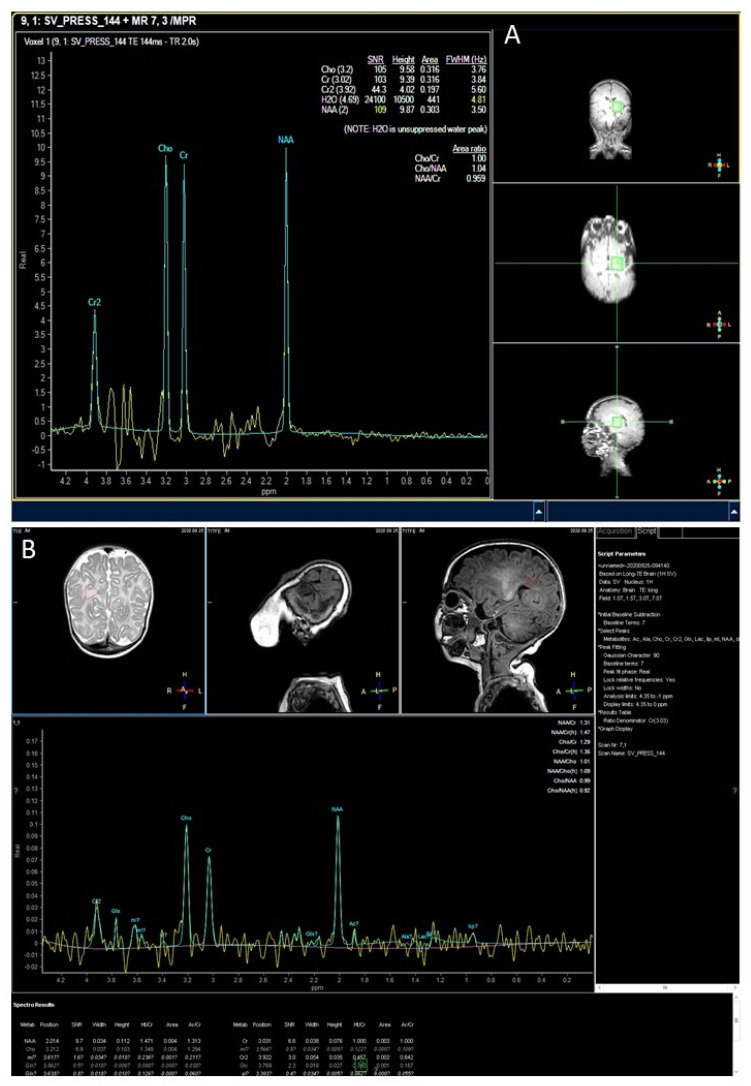
MR Spectroscopy of Patients 2 and 4. MR spectroscopy of Patient 2 showing almost equally high peaks of choline, creatine, and N-acetyl-aspartate, characteristic of leukoencephalopathy and hypomyelination (**A**). Near-normal MR spectrogram in a less advanced stage of patient 4 (**B**).

Figure 2 presents the MR spectroscopy results of Patients 2 and 4. MR spectroscopy of Patient 2 shows almost equally high peaks of choline, creatine, and N-acetyl-aspartate, characteristic of leukoencephalopathy and hypomyelination (Figure 2A). Near-normal MR spectrogram is seen in a less advanced stage in Patient 4 (Figure 2B).

### 3.3. Results of the Genetic Testing

In the promoter region of the *UFM1* gene, we have found the c.-155_-153delTCA mutation in homozygous form in four apparently unrelated index patients and in heterozygous form in a further three family members.

**Figure 3:**
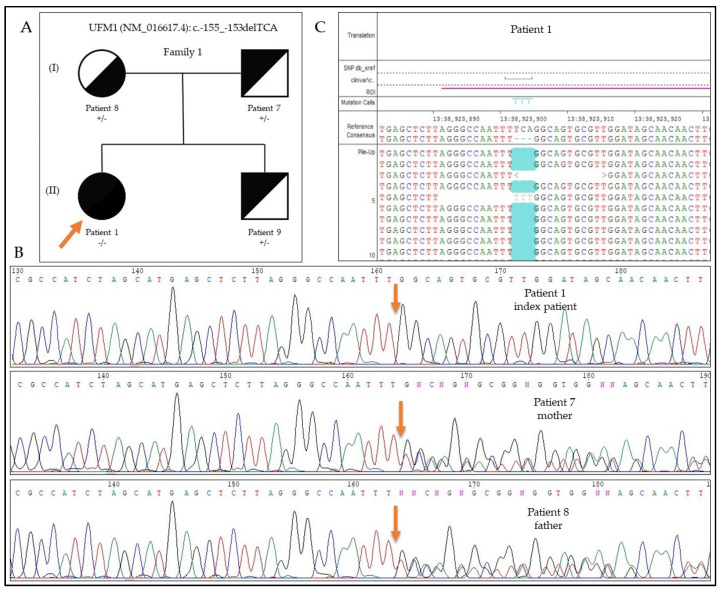
Pedigree and sequencing results of Family 1. (**A**) Family tree of Family 1. (**B**) Sanger sequencing of Patients 1, 7, and 8. Patient 1 is homozygous; Patients 7 and 8 are heterozygous for the *UFM1*: c.-155_-153delTCA mutation. (**C**) Next-generation sequencing results of Patient 1, showing the homozygous deletion.

The sequencing results of Family 1 are presented in Figure 3. Patient 1 is homozygous for the *UFM1* c.-155_-153delTCA mutation (WGS with a coverage of 22×), whereas both parents are carrying the mutation in a heterozygous form. Patient 1 has a younger, unaffected brother, who also carries the mutation in a heterozygous form (as judged by Sanger sequencing). Parents were available for testing only from this family.

**Figure 4:**
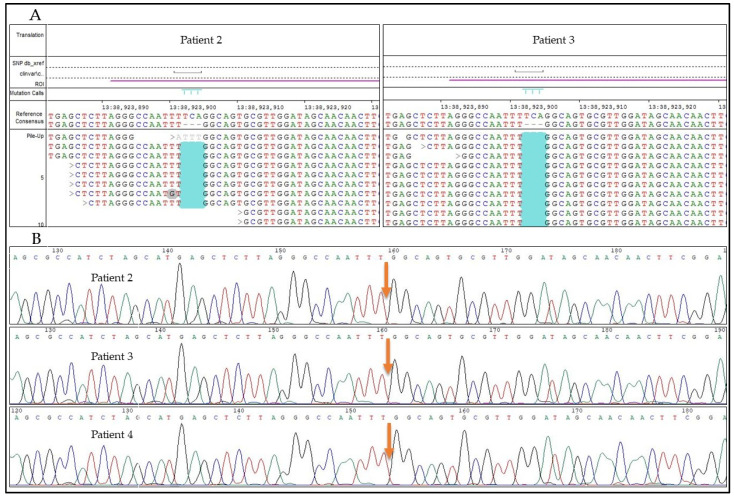
Next-generation sequencing (**A**) results of Patients 2 and 3. Sanger sequencing (**B**) results of Patients 2, 3, and 4. All three patients are homozygous for the *UFM1*: c.-155_-153delTCA mutation.

Patients 2 and 3 are also homozygous for the *UFM1*: c.-155_-153delTCA mutation (presented in Figure 4), with a coverage of 7× and 27× (WES), respectively. 

Patient 4 (Figure 4), who showed a very similar clinical course to the patients previously identified, was identified in a Sanger sequencing setting targeted for the *UFM1*: c.-155_-153delTCA founder mutation and resulted to be homozygous as well.

## 4. Discussion

Hypomyelinating leukodystrophy is a genetically heterogeneous disorder of the central nervous system in which myelin is not formed properly [6]. To date, about 20 different types are distinguished, one of which is Hypomyelinating leukodystrophy type 14 (MIM # 617899), caused by mutations in the *UFM1* gene. It is characterized by hypotonia, almost complete lack of motor or cognitive skills, absent language development, spasticity, and intractable seizures. Most patients die in the first few years of life [7]. It is inherited in an autosomal recessive manner.

The *UFM1* (NM_016617.4) c.-155_-153delTCA mutation present in the promoter region of the gene is reported to be a founder mutation in the Roma population, with a carrier frequency of 3–25%. This deletion has a neural cell-specific effect of a significant reduction in transcription activity [7]. It should be noted that the c.-155_-153delTCA mutation was found in two unrelated samples arriving at the laboratory for diagnostic WES from patients with clinical phenotype other than hypomyelinating leukodystrophy as the incidental finding.

Here we presented four patients with homozygous *UFM1*:c.-155_-153delTCA mutation, detected by next-generation sequencing (WES/WGS) or Sanger sequencing. Ethnicity was assessed retrospectively in three families, and in the patient from the fourth family—along with the brain MRI and clinical course—it served as an important pillar of the diagnostic workup in which targeted Sanger sequencing of the *UFM1* founder mutation led to a fast molecular diagnosis. We could conclude that all patients homozygous for the *UFM1*:c.-155_-153delTCA mutation were found in the Roma population. Only the parents in Family 1 were accessible for testing.

When assessing the clinical features, we found that only one of our patients had epilepsy, but they all had EEG abnormalities. Painful tonic fits and clonisation of the lower limbs were seen in almost all of them, giving the impression of seizures, but the electroencephalograms showed diffuse cortical dysfunction instead. One patient developed epilepsy and was treated accordingly. Altered consciousness manifesting as somnolence, stupor, or irritability were seen in all patients. All but the youngest patient had life-threatening vegetative symptoms that eventually led to their death, including severe bradypnea with or without bradycardia. In one patient, a mildly low body temperature was registered. The median survival of the patients was 28 months, not counting the youngest who is still alive at 17 months of age. It is difficult to assess regression in young children with almost no psychomotor development at all, where intentional movements were never achieved and cognition can only be judged based on their visual tracking and alertness, but considering that even their minimal attention and alertness diminished; regression eventually can be stated. We also noticed that abnormal muscle tone not only affected skeletal muscles but apparently the laryngeal muscle, causing loud stridor, jugular retraction, and ineffective inhalation phase during breathing; therefore, most patients needed a tracheostomy. Respiratory failure was repeatedly described by Nahorski et al. and Hamilton et al. [5,7], but we would like to draw attention to the abnormal pattern of breathing in the advanced stage of the disease.

Brain atrophy and hypomyelination, predominantly affecting the caudate nuclei and cerebral cortex, were seen in all patients; this was relatively mild in Patient 4, which may be due to her early age at the time of the clinical and neuroradiological evaluation and a less advanced stage of the disease. In this patient, we were able to apply a targeted single mutation testing for the *UFM1* founder mutation based on the strong clinical clues, suspected inheritance, and ethnic background. To our knowledge, there is no literature report of targeted testing for the *UFM1* founder mutation instead of a panel testing or WES; we have been the first ones to do it in the case of an infant with Hypomyelinating leukodystrophy, and this lead to a faster molecular genetic diagnosis of the patient.

As the coverage of the promoter region of the *UFM1* gene is generally poor in the case of exome sequencing data, and in many cases only the coding region of the genes are analyzed, this pathogenic founder variant could be easily missed; therefore, diagnostic laboratories should pay special attention to this region.

By adding the phenotypic and molecular data of a further four patients to the 19 described to date [7,8,9,10], we aimed to expand the clinical and molecular knowledge on this rare entity. We did not see a geographical accumulation of the patients within Hungary; they originated from different parts of the country. We also conclude that targeted testing of the *UFM1*:c.-155_-153delTCA mutation may provide an indication in cases of hypomyelination and neurodegenerative clinical course in pediatric patients of Roma descent. Finally, a large population screening is warranted in the potentially affected Roma populations to analyze carrier frequency in order to offer preconceptional targeted genetic testing for the affected couples.

## Figures and Tables

**Table 1:** Zygosity of individuals for the *UFM1*:c-155_-153delTCA mutation. P7, P8, and P9 were tested in frames of cascade testing. Only the parents of P1 were available for testing. All patients are of Roma descent.

Family	Sample	Zygosity
F1	P1	Homozygous
F1	P7 (mother)	Heterozygous
F1	P8 (father)	Heterozygous
F1	P9 (sibling)	Heterozygous
F2	P2	Homozygous
F3	P3	Homozygous
F4	P4	Homozygous

**Table 2:** Clinical characteristics of patients homozygous for the *UFM1* c.-155_-153delTCA mutation. The table is based on and expands the work of Nahorski et al., 2018 [5]. N/A—not assessed.

Clinical Features	P1	P2	P3	P4	Total
General					
Gender	F	M	M	F	1:1 ratio
Age of onset/medical attention drawn	2 weeks	1 week	6 months	3.5 months	Average months 2.5
Growth					
Failure to thrive	Yes	Yes	Yes	No	3/4
Short stature	No	Yes	Yes	No	2/4
Microcephaly	Yes	Yes	Yes	No	3/4
Central nervous system					
Global developmental delay (severe)	Yes	Yes	Yes	Yes	4/4
Intellectual disability (profound)	Yes	Yes	Yes	Yes	4/4
Axial hypotonia	Yes	N/A	Yes	Yes	3/4
Spasticity	Yes	Yes	Yes	Yes	4/4
Regression	Yes	Yes	Yes	Yes	4/4
Seizures	No	Yes	No	No	1/4
Abnormal EEG (diffuse cortical dysfunction)	Yes	Yes	Yes	Yes	4/4
Brain atrophy (cortex/ basal ganglia)	Yes	Yes	Yes	Yes	4/4
Delayed/absent myelination	Yes	Yes	Yes	Yes	4/4
Cerebellar hypoplasia	Yes	Yes	Yes	Yes	4/4
Vegetative functions					
Feeding difficulty	Yes	Yes	Yes	No	3/4
Laryngeal stridor	Yes	Yes	Yes	No	3/4
Bradypnea	Yes	Yes	Yes	No	3/4
Bradycardia	Yes	No	N/A	No	1/4
Altered levels of consciousness (somnolence, stupor, irritability)	Yes	Yes	Yes	Yes	4/4
Age of death (months)	17	38	30	Alive at 17 months	Median survival 28 months

## Data Availability

The data presented in this study are available on request from the corresponding author.
